# From Bad to Worse: Paraganglioma Diagnosis during Induction of Labor for Coexisting Preeclampsia

**DOI:** 10.1155/2017/5495808

**Published:** 2017-01-18

**Authors:** Sasima Dusitkasem, Blair H. Herndon, Dalton Paluzzi, Joseph Kuhn, Robert H. Small, John C. Coffman

**Affiliations:** Department of Anesthesiology, The Ohio State University Wexner Medical Center, Columbus, OH, USA

## Abstract

Pheochromocytomas and extra-adrenal paragangliomas are catecholamine-secreting tumors that rarely occur in pregnancy. The diagnosis of these tumors in pregnancy can be challenging given that many of the signs and symptoms are commonly attributed to preeclampsia or other more common diagnoses. Early diagnosis and appropriate management are essential in optimizing maternal and fetal outcomes. We report a rare case of a catecholamine-secreting tumor in which diagnosis occurring at the time labor was being induced for concomitant preeclampsia with severe features. Her initial presentation in hypertensive crisis with other symptoms led to diagnostic workup for secondary causes of hypertension and led to eventual diagnosis of paraganglioma. Obtaining this diagnosis prior to delivery was essential, as this led to prompt multidisciplinary care, changed the course of her clinical management, and ultimately enabled good maternal and fetal outcomes. This case highlights the importance of maintaining a high index of suspicion for secondary causes of hypertension and in obstetric patients and providing timely multidisciplinary care.

## 1. Introduction

Pheochromocytomas and extra-adrenal paragangliomas (PGLs) are catecholamine-secreting tumors that arise from neural-crest derived chromaffin cells [1]. These rare tumors are estimated to occur in 0.1–0.2% of hypertensive adults [2, 3] and 0.002% of pregnant women [4, 5]. Extra-adrenal PGLs have been estimated to account for 19% of chromaffin cell tumors occurring in pregnancy, with pheochromocytomas accounting for the majority of tumors [6]. The classical presentation is associated with episodic catecholamine secretion by the tumor, resulting in paroxysmal hypertension, headaches, sweating, and palpitations [7, 8]. The diagnosis of catecholamine-secreting tumors in pregnancy is challenging given that many of these signs and symptoms are commonly attributed to preeclampsia or other diagnoses that occur more commonly in pregnancy [7–9]. However, it is important to maintain a high level of suspicion for pheochromocytoma or PGL in cases of severe hypertension refractory to conventional antihypertensive treatment, as early diagnosis and appropriate treatment can improve maternal and fetal outcomes [6].

We report an undiagnosed paraganglioma (PGL) in a pregnant woman at 33 weeks of gestation who presented with hypertensive crisis. This complex case presented a unique diagnostic challenge given that the patient had coexisting preeclampsia, though timely diagnosis changed the course of her clinical management and ultimately enabled positive maternal and fetal outcomes.

## 2. Case Presentation

A 33-year-old G6P3 woman (weight 83.9 kg; height 1.6 m; body mass index 32.8 kg/m^2^) presented to our unit at 32 5/7 weeks of gestation with severe headache, palpitations, anxiety, abdominal pain, and hypertensive crisis (blood pressure (BP) 200–230/100–130 mmHg). Her past medical history was significant for viral myocarditis with subsequent cardiomyopathy three years previously. At that time, her left ventricular ejection fraction (LVEF) was <20%, and she required extracorporeal membrane oxygenation (ECMO). She was decannulated after normalization of her ejection fraction (LVEF 73%). After decannulation, she developed hypertension and ultimately required initiation of four antihypertensive medications prior to hospital discharge. With hypertension at this level of severity in a 30-year-old woman, a workup for secondary causes of hypertension should have been initiated.

She did not follow up with scheduled outpatient appointments and was lost to follow-up until the current pregnancy. Unfortunately, a more detailed workup for etiologies of her severe hypertension was not completed during the patient's first and only prenatal visit at 14 weeks of gestation. She was no longer taking any antihypertensives prior to this visit, and labetalol 100 mg twice daily was initiated as blood pressure was noted to be 158/72 mmHg. She did not endorse symptoms of headache, palpitations, temperature intolerance, or recent weight changes at that time. She did not attend her other obstetric appointments and was noncompliant with her labetalol.

Upon hospital presentation, she was diagnosed with chronic hypertension with superimposed preeclampsia based on a 24-hour urine protein of 388 mg. Intravenous hydralazine 10 mg and labetalol 20 mg were given for initial control of her BP. She was admitted to the antepartum unit and maintained on oral labetalol 200 mg twice daily for BP control and received betamethasone to enhance fetal lung maturation. Her BP control improved, though she continued to experience intermittent headaches. Urinary and serum metanephrines were collected given the severity of hypertension accompanied by headaches and palpitations, though there was only mild suspicion at this point in time. These samples are processed by an external laboratory which, due to transit times, consequentially causes a delay in the availability of results. Seven days after admission for preeclampsia, labor was induced due to worsening hypertension and headache symptoms. Though the secondary workup had been initiated, the working diagnosis was still superimposed preeclampsia with severe features that could no longer be controlled by medications. Given that catecholamine-secreting tumors were less likely among differential diagnoses at that time, no alpha or calcium channel blockade was initiated prior to induction of labor (IOL). Intravenous magnesium 4 gm was given, followed by IV infusion of magnesium 2 gm/hr. She had an uneventful epidural placement at L3-4 and obtained effective epidural labor analgesia after an initial dose of 0.125% bupivacaine 10 mL and fentanyl 20 mcg. Labor analgesia was then maintained with a continuous epidural infusion of 0.0625% bupivacaine + 2 mcg/mL fentanyl at 12 mL/hr, with patient-controlled epidural analgesia (8 mL bolus, 15-minute lockout). During her induction, her free plasma and 24-hour urinary and metanephrines and normetanephrines resulted and were found to be significantly elevated (see Table 1). Despite cervical dilation to 5 cm and artificial rupture of membranes, her IOL was stopped and a multidisciplinary meeting consisting of obstetricians, anesthesiologists, endocrinologists, and surgical oncologists was promptly convened to develop appropriate treatment plan. After discussion with the various care teams, a cesarean delivery was planned. Cesarean was determined to be the optimal mode of delivery to avoid rises in intraabdominal pressure and catecholamine releases associated with uterine contractions and vaginal delivery. Due to continued hypertensive urgency, cervical dilation, and ruptured membranes, delivery proceeded in an expedited fashion once patient has been dosed with phenoxybenzamine 10 mg and volume replete with intravenous fluids. The recommendation from the multidisciplinary meeting was to not remove the mass at time of cesarean delivery, as the patient had not been alpha-blocked for a significant amount of time and manipulation of the mass could cause significant morbidity.

A radial arterial line was placed preoperatively for close monitoring of blood pressure. Appropriate emergency medications were prepared for intraoperative administration, including IV phentolamine and nicardipine. IV magnesium 2 gm/hr was continued intraoperatively for prevention of eclamptic seizures and to help manage intraoperative hypertension due to her catecholamine-secreting tumor. Her labor epidural was effectively dosed for cesarean anesthesia with 0.5% ropivacaine 20 mL, given in 3–5 mL increments over fifteen minutes, and fentanyl 100 mcg. Her vital signs were stable during the initial operative course, but she became acutely hypertensive to >220/110 with external abdominal pressure during delivery. This was effectively managed with IV nicardipine 500 mcg in incremental doses. Nicardipine 5 mg/hr was initiated toward the end of the procedure and continued for 18 hours. The operation was successful; a 2,570 g female newborn was delivered, with neonatal Apgar scores of 4 at one minute, 3 at five minutes, and 7 at ten minutes. Umbilical arterial pH was 7.28 and umbilical venous pH was 7.36.

**Figure 1:**
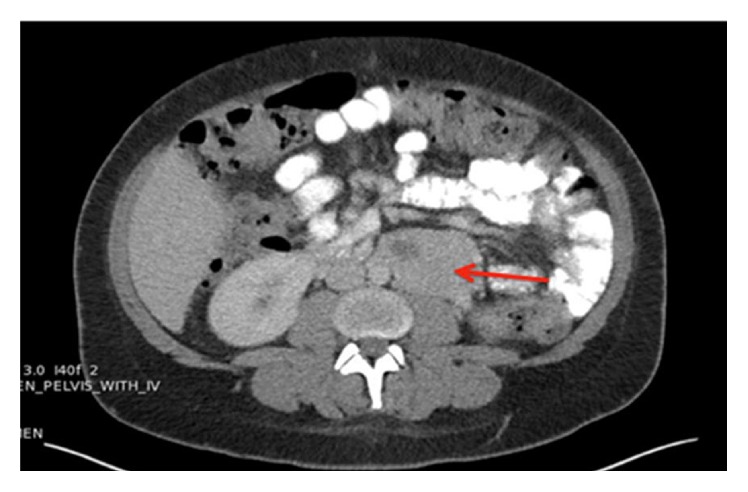
CT abdomen/pelvis demonstrating a left para-aortic mass measuring approximately 4.7 × 6.4 cm.

The patient was observed in the intensive care unit postoperatively. Her postpartum period was uneventful. CT imaging revealed a left para-aortic mass consistent with PGL (Figure 1). During her ICU stay, oral phenoxybenzamine 10 mg twice daily (BID) and propranolol 10 mg every 8 hours were initiated. Endocrine specialists gradually increased phenoxybenzamine to 60 mg every 8 hours and propranolol to 60 mg every 8 hours, over a two-week period of medical optimization prior to surgical resection of the paraganglioma. An arterial line was placed for careful hemodynamic monitoring. Anesthesia was induced after 4 mg of midazolam with 2% lidocaine 100 mg, propofol 200 mg, fentanyl 200 mcg, and 50 mg rocuronium. Smooth tracheal intubation followed, during which blood pressures peaked at 210/130. This required treatment with 600 mcg of nicardipine given in divided doses. As the surgeons dissected to the tumor, phenylephrine was needed to maintain blood pressure. Pressures reached a nadir of 90/40. Upon manipulation and dissection of the mass, hemodynamics became extremely labile despite the 2-week adrenergic receptor blockade. Intraoperative hemodynamic stability was achieved by careful fluid management, administration of incremental boluses of nicardipine, and esmolol, as well as nitroprusside and remifentanil infusions. Upon removal of the tumor, hemodynamics stabilized and the patient was able to be weaned off the infusions. She required some minimal blood pressure support with phenylephrine during closure. The tumor specimen pathology examination revealed features consistent with the diagnosis of extra-adrenal paraganglioma. She was discharged along with a healthy neonate seven days later without complication. Labs one month after resection showed normalization of her plasma normetanephrine and metanephrine levels (see Table 1). The patient has not been compliant with scheduled follow-up appointments with endocrine, genetics, obstetrics, or surgical oncology specialists.

## 3. Discussion

Maternal and fetal outcomes in patients with catecholamine-secreting tumors are greatly improved by early antenatal diagnosis and proper management with alpha-adrenergic blockade and sometimes antenatal surgical resection of the tumor [5, 6]. Biggar and Lennard reported that early antenatal recognition of pheochromocytoma/PGL resulted in decreased maternal mortality from 29% to 0% and fetal mortality from 29% to 12% compared with diagnoses made during labor or postpartum [6]. Higher incidence of maternal and fetal mortality has been observed with pheochromocytoma (9.8% and 16%, resp.) compared to extra-adrenal PGL (3.6 and 12%, resp.) in obstetric patients [4], though the tumor location in our case was not determined until after delivery. Regardless of the chromaffin cell tumor location, high maternal, and fetal morbidity and mortality rates underscore the importance of establishing a diagnosis as early as possible. While poor medical compliance of our patient prior to and during pregnancy limited the opportunity of antenatal tumor diagnosis, it is true that hypertension this severe in a young person should have been worked up previously. The lack of workup for 4-drug resistant hypertension after our patient's ECMO decannulation, or during her single prenatal visit, significantly increased mortality risks. She was diagnosed with chronic hypertension with superimposed preeclampsia due to the presence of proteinuria, which is not commonly associated with pheochromocytoma or PGL [5, 6]. Importantly, the physicians involved in her hospital care also searched for secondary causes of hypertension based on modest clinical suspicion given her initial presentation of hypertensive urgency accompanied by headache, sweating, palpitations, and other symptoms. Diagnostic workup revealed the eventual diagnosis of a catecholamine-secreting tumor, which led to prompt multidisciplinary care, altered the course of her clinical management, and ultimately enabled good maternal and fetal outcomes. This case highlights the importance of maintaining a high index of suspicion for secondary causes of hypertension in obstetric patients.

It was essential to have timely involvement and good communication among the obstetricians, anesthesiologists, endocrinologists, and surgical oncologists involved in her care. Based on multidisciplinary discussions, it was decided that cesarean delivery would be the optimal course of action. Obstetric patients with pheochromocytoma or PGL have had successful vaginal deliveries, though cesarean deliveries are more commonly performed [4, 6]. Higher fetal mortality rates have been observed with vaginal delivery compared to cesarean delivery in this setting [10]. The fetus is not directly exposed to the high catecholamine levels due to enzymatic metabolism within the placenta [11], though uncontrolled hypertension can lead to uteroplacental insufficiency, placental abruption, and fetal demise. Hypertension due to catecholamine release may be precipitated by increases in intra-abdominal pressure during uterine contractions, vigorous fetal movements, or second stage pushing with vaginal delivery [12–15]. In addition to potential mechanical disturbance of the tumor, the stresses of labor pain can induce sympathetic stimulation and catecholamine release [6]. Further, this patient was diagnosed during the course of labor and had not been medically optimized on adrenoceptor blockade, which is important prior to both tumor resection and any other procedure that may be associated with catecholamine release from the tumor [16, 17]. Based on these reasons, the multidisciplinary team of physicians caring for this patient agreed that cesarean delivery with delayed surgical resection was the most appropriate course of action. The optimal time for surgical tumor removal is controversial and depends in part upon the gestational age at the time of diagnosis. Tumors can be removed either before 24 weeks of gestation, simultaneously with cesarean delivery, or separately after delivery [18]. In our patient, surgical excision was performed 2 weeks postpartum after inpatient medical stabilization, as there was concern regarding patient compliance as an outpatient.

Undiagnosed chromaffin cell tumors can present with life-threatening complications for both the mother and fetus. Patients presenting with hypertensive crisis, as our patient did, have been reported to have maternal mortality rates of 54.5% [6]. This finding highlights the importance of early diagnosis and also maintaining control of hypertension with adrenoceptor blockade. Preoperative BP control is essential to avoid hypertensive crisis during cesarean delivery or surgical resection of the tumor. Phenoxybenzamine is an irreversible adrenergic antagonist and has been suggested as the preferred treatment of choice in patients with pheochromocytoma or PGL [19]. Our patient was started on phenoxybenzamine for alpha blockade and labetalol was discontinued. Although labetalol has both alpha and beta blocking properties, the ratio of alpha : beta antagonism is approximately 1 : 7 and could lead to unopposed alpha-adrenergic receptor stimulation [20]. Beta-blockade with propranolol was initiated more than 24 hours after treatment with phenoxybenzamine in order to minimize a paradoxical hypertensive response.

The primary goal of her intraoperative anesthetic management for cesarean delivery was to prevent and effectively manage abrupt changes in her hemodynamic measurements. Neuraxial anesthesia was desirable in this patient with PGL given the potential advantage of avoiding hypertension and tachycardia due to sympathetic stimulation and catecholamine secretion associated with laryngoscopy, intubation, and surgical stress [21, 22]. Epidural blockade was gradually induced as IV crystalloid was administered and arterial line BP carefully monitored in order to avoid hypotension and maintain appropriate placental blood flow. Nicardipine was used effectively for treatment of severe hypertension at the time of delivery [23, 24] and was also effectively utilized in the early postdelivery period. Magnesium sulfate was also useful in her hemodynamic management, where the patient was getting this for concomitant preeclampsia. Magnesium sulfate inhibits catecholamine release from the catecholamine-secreting tumor and blocks peripheral catecholamine receptors [9].

After tumor resection, it is important to check plasma or urinary metanephrine levels 2–6 weeks to assess for completeness of resection [25]. It is also important to continue regular surveillance with endocrine specialists, given that tumor persistence or new tumors can occur. For example, 18% of patients with thoraco-abdomen-pelvic PGLs have been reported to have a new tumor event within the first five years after initial surgical resection [25]. Referral for genetic testing is also essential given that a germline mutation in susceptibility genes is identified in approximately 40% of pheochromocytomas or paragangliomas cases [26], which can increase risk of tumor recurrence and also have implications for family members [25]. Unfortunately, the patient described in this case has not arrived for scheduled appointments with endocrine or genetics specialists up to this point.

In conclusion, this case presented a unique diagnostic challenge given that the patient had coexisting preeclampsia, which can share many of the same signs and symptoms as catecholamine-secreting tumors. Diagnosis prior to the patient's IOL would have been ideal, as the patient could have been optimized with alpha adrenergic blockade prior to delivery. Fortunately, multidisciplinary collaboration and planning allowed for a mode of delivery and ultimate tumor resection that enabled good maternal and fetal outcomes.

## Figures and Tables

**Table 1:** The results of free plasma and 24-hour urinary and metanephrines and normetanephrines.

	Reference values	Values at 33-week gestation	Values 1 month after tumor excision
Urinary normetanephrines (mcg/24 hrs)	111–419	**11617**	
Urinary metanephrine (mcg/24 hrs)	30–180 (normotensive) <400 (hypertensive)	291	
Total urinary metanephrines (mcg/24 hrs)	149–535 (normotensive) <1300 (hypertensive)	**11908**	
Plasma normetanephrines (pg/mL)	<148	**2920**	110
Plasma metanephrines (pg/mL)	<57	<**25**	41
